# UAS™—A Urine Preservative for Oncology Applications

**DOI:** 10.3390/cancers15123119

**Published:** 2023-06-08

**Authors:** Stephanie Jordaens, Amit Arora, Kyle W. MacDonald, Cameron Wood, Jhana O. Hendrickx, Karen Zwaenepoel, Christophe Deben, Wiebren Tjalma, Patrick Pauwels, Koen Beyers, Vanessa Vankerckhoven

**Affiliations:** 1 Center for Oncological Research (CORE), Integrated Personalized & Precision Oncology Network (IPPON), University of Antwerp, 2610 Wilrijk, Belgium; karen.zwaenepoel@uza.be (K.Z.); christophe.deben@uantwerpen.be (C.D.); wiebren.tjalma@uza.be (W.T.); patrick.pauwels@uantwerpen.be (P.P.); 2 Novosanis NV, 2110 Wijnegem, Belgium; jhana.hendrickx@novosanis.com (J.O.H.); koen.beyers@novosanis.com (K.B.); vanessa.vankerckhoven@uantwerpen.be (V.V.); 3 DNA Genotek Inc., Ottawa, ON K2V 1C2, Canada; amit.arora@dnagenotek.com (A.A.); kyle.macdonald@dnagenotek.com (K.W.M.); cameron.wood@dnagenotek.com (C.W.); 4 Laboratory of Pathological Anatomy, Antwerp University Hospital (UZA), 2650 Edegem, Belgium; 5 Multidisciplinary Breast Clinic, Gynecological Oncology Unit, Department of Obstetrics and Gynecology, Antwerp University Hospital (UZA), 2650 Edegem, Belgium; 6 Vaccine & Infectious Disease Institute (VAXINFECTIO), Faculty of Medicine and Health Sciences, University of Antwerp, 2610 Wilrijk, Belgium

**Keywords:** oncology, liquid biopsy, first-void urine, urine collection, urine preservation, cancer, carcinoma, detection, screening, secondary prevention, treatment, response, breast, prostate, pregnancy

## Abstract

**Simple Summary:**

In the field of cancer, body fluids such as urine are gaining interest for non-invasive cancer detection and screening. To enable home collection, sample shipment, and sample storage at ambient temperatures, the addition of preservatives is critical. In this study, we evaluated the performance of a recently developed preservative, UAS™, for the preservation of urinary analytes in oncology applications. We demonstrated that UAS™ preserves host cell integrity and cell-free DNA and prevents bacterial overgrowth under conditions encountered with remote collections. Additionally, UAS™ is compatible with commercially available urinary analyte isolation kits. The preservative facilitates urine collection in the comfort and privacy of the patient’s home, making cancer screening programs more accessible and lowering the burden on healthcare workers.

**Abstract:**

Liquid biopsy is a revolutionary tool that is gaining momentum in the field of cancer research. As a body fluid, urine can be used in non-invasive diagnostics for various types of cancer. We investigated the performance of UAS™ as a preservative for urinary analytes. Firstly, the need for urine preservation was investigated using urine samples from healthy volunteers. Secondly, the performance of UAS™ was assessed for cell-free DNA (cfDNA) and host cell integrity during storage at room temperature (RT) and after freeze-thaw cycling. Finally, UAS™ was used in a clinical setting on samples from breast and prostate cancer patients. In the absence of a preservative, urinary cfDNA was degraded, and bacterial overgrowth occurred at RT. In urine samples stored in UAS™, no microbial growth was seen, and cfDNA and cellular integrity were maintained for up to 14 days at RT. After freeze-thaw cycling, the preservation of host cell integrity and cfDNA showed significant improvements when using UAS™ compared to unpreserved urine samples. Additionally, UAS™ was found to be compatible with several commercially available isolation methods.

## 1. Introduction

Cancer is one of the leading causes of death, with an estimated 19.3 million new cases and 10 million deaths worldwide in 2020 [1]. Liquid biopsy is a revolutionary method that has been gaining momentum in the past decades in the field of cancer research and care and has important advantages over tissue biopsy. Liquid biopsy can capture intratumor heterogeneity, while tissue biopsy only offers a snapshot of the tumor at a specific location and at a given time. Different body fluids, including blood, cerebrospinal fluid, saliva, sputum, and urine, have great potential as liquid biopsies [2,3,4,5]. Liquid biopsies can be used for cancer screening and therefore (i) have the potential to significantly impact the early detection and prognosis of cancer patients, (ii) will allow for monitoring of treatment response, and (iii) will allow for detection of recurrence [2,3,4,5]. Moreover, these liquid biopsies can be obtained using minimally invasive (blood, cerebrospinal fluid) or fully non-invasive procedures (urine, saliva, sputum), offering the possibility to sample more frequently and monitor the patient closely. Lately, urine as a liquid biopsy is gaining interest for non-invasive cancer diagnosis for different cancer types [2,3,4,5], including but not limited to urological cancers, breast cancer, and lung cancer [6]. Due to the non-invasive nature of urine collection, it is easy to collect even at home, eliminating the need for collection at the clinic. It can be collected repeatedly and without the volume restrictions often incurred with other liquid biopsy sample types, allowing for serial sampling and multi-omics analysis. Human urine is a complex biological fluid that primarily consists of water, organic and inorganic compounds, bacteria, and multiple cell types, including erythrocytes, leukocytes, prostate cells, renal cells, and urothelial cells [4]. Cellular and cell-free material can be released directly into the urine or via glomerular filtration [7]. Our previously conducted literature review on urinary biomarkers has shown that urine contains several analytes, including DNA, RNA, extracellular vesicles (EVs), proteins, and metabolites [6]. These analytes make urine an attractive multi-omic sample type, potentially allowing for genomics, epigenomics, transcriptomics, proteomics, metabolomics, and microbiome analysis [2,3,4,5].

Urinary cell-free DNA (cfDNA) exists in the extracellular space and can be classified into two categories according to their size: low-molecular-weight (LMW) DNA (10–250 bp) and high-molecular-weight (HMW) DNA (1 kb or higher). LMW DNA can be derived from apoptotic cells in urine or circulation via glomerular filtration, and HMW DNA can originate from necrosis of cells shedding from the urogenital tract directly into the urine. Cellular DNA (in kbs) resides in the urogenital tract cells or debris shedding into the urine [7,8,9,10,11]. Urinary DNA can be investigated for tumor-specific changes, including methylation, microsatellite composition disorders, and point mutations, as well as the presence of viral DNA [9,12,13].

Urine is a promising emerging liquid biopsy; however, guidelines around pre-analytical parameters, including collection, preservation, and storage, are not yet well defined. This study investigated the important issue of the need for standardization of preanalytical protocols and the importance of urine preservation for oncological applications in light of a home collection and screening setup. This aligns with earlier studies [12,13,14], including our recent publication [6]. Here, we investigated the performance of a recently developed preservative, UAS™ [15], for the preservation of urinary cfDNA and host cell integrity at RT for up to 14 days and after freeze-thaw cycling for oncology applications. Thereafter, this preservative was used in a clinical setting on samples from breast and prostate cancer patients.

## 2. Materials and Methods

### 2.1. Pre-Evaluation of the Need for Urine Preservation

Samples were collected under a protocol approved by institutional review board provider Advarra (Columbia, WA, USA), and informed consent was obtained from participants prior to sample collection. Unpreserved, first-void urine samples were obtained from five females and two males using a standard urine collection cup or Colli-Pee^®^ FV-5020 (Novosanis, Wijnegem, Belgium). Samples were transported to the lab on ice packs within 5–6 h of collection. Each urine sample was aliquoted (4.5 mL per aliquot); one aliquot was taken as a baseline sample (T0), and the other was stored at RT (20–26 °C) for seven days (T7). At both time points, the aliquots were centrifuged at 3800× *g* for 20 min. The obtained supernatants (4 mL) were stored at −80 °C for downstream processing. Total cell-free nucleic acids (cfTNA) were extracted using the QIAamp^®^ Circulating Nucleic Acid Kit (Qiagen, Hilden, Germany). The extracted cfTNA was analyzed using a 16S bacterial rDNA qPCR and a human β-globin DNA qPCR assay (see below). Additionally, the sample DNA profile was investigated using the High-Sensitivity D5000 DNA ScreenTape (Agilent, Santa Clara, CA, USA) on the Agilent 4150 TapeStation system.

### 2.2. Evaluation of Urine Storage Conditions with UAS™ Preservative

#### 2.2.1. Sample Collection

Samples were collected under a protocol approved by institutional review board provider Advarra (Columbia, WA, USA), and informed consent was obtained from participants prior to sample collection. First-void urine samples were obtained from 42 females and 42 males using Colli-Pee^®^ FV-5040 (Novosanis, Wijnegem, Belgium) without preservatives. The urine samples were collected between 10 a.m. and 1 p.m., and donors were instructed to wait a minimum of one hour after their last urination.

#### 2.2.2. Experimental Set-Up

**Figure 1:**
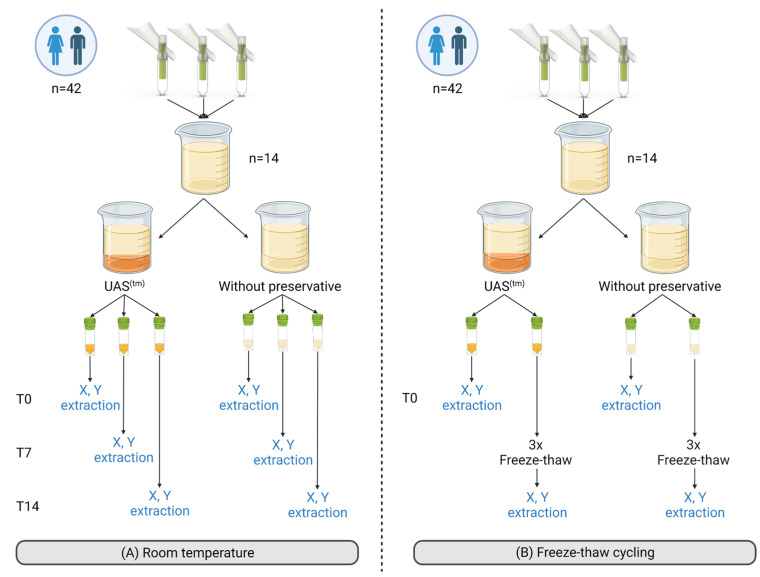
Experimental set-up to evaluate UAS™ preservative performance under different storage conditions—Sample collection and pooling were done similarly for the room temperature experiment (**A**) or the freeze-thaw experiment (**B**); urine samples were obtained from 21 females and 21 males using Colli-Pee^®^ FV-5040 (Novosanis) without preservative. After collection, three female or male samples were combined to form female pooled (FP) or male pooled (MP) urine samples, respectively. A total of 14 (7 FP and 7 MP) pooled urine samples were generated and used for the experiment. Abbreviations: no addition, neat; T0, day 0 or baseline; T7, 7 days; T14, 14 days; X, cell-free total nucleic acid extraction; Y, cellular pellet extraction. Created with BioRender.com.

Three female or male samples were combined to form female-pooled (FP) or male-pooled (MP) urine samples, respectively. A total of 28 (14 FP and 14 MP) pooled urine samples were generated. A total of 14 pooled urine samples (7 FP and 7 MP) were used for RT (20–26 °C) and freeze-thaw cycling testing (Figure 1). After pooling, each pooled sample was split into two aliquots (55 mL total), with the UAS™ preservative being added to the first aliquot (38.5 mL urine with 16.5 mL UAS™ preservative), while the other was left as is without preservative addition (neat, 55 mL unpreserved urine).

1. UAS™ preservative performance during storage at room temperature

UAS™ preserved (55 mL) and neat (55 mL) pooled urine samples were aliquoted separately into three aliquots (18 mL per aliquot). One aliquot of each pooled sample was processed immediately at baseline (T0), while the remaining aliquots were stored at RT (20–26 °C) for 7 (T7) or 14 days (T14) before processing (Figure 1).

2. UAS™ preservative performance after freeze-thaw cycling

UAS™ preserved (55 mL) and neat (55 mL) pooled urine samples were separated into two aliquots (20 mL per aliquot). One aliquot of each pooled sample was processed immediately at baseline (T0), while the other aliquot underwent 3 freeze-thaw cycles (−20 °C to +40 °C) with a minimum of 3 h at each temperature per cycle before processing (Figure 1).

#### 2.2.3. Extractions

- Sample processing

After the specified conditions, each aliquot was centrifuged at 3000× *g* for 10 min. The resulting supernatant was filtered through a 0.8 µm filter into a new 50-mL conical tube and stored at −80 °C for downstream extraction. The resulting pellet was resuspended in 100 µL of PBS, transferred to a 1.5 mL Eppendorf tube, and stored at 80 °C for downstream extraction.

- Cell-free total nucleic acid extraction

Samples were extracted in batches such that all corresponding aliquots (baseline, time points, or post-freeze-thaw cycling) from a given pooled sample were extracted at the same time. Samples were removed from the −80 °C freezer and thawed in a 37 °C water bath for approximately 15 min. cfTNA was extracted using the QIAamp^®^ Circulating Nucleic Acid Kit (Qiagen, Hilden, Germany). For the urine samples containing UAS™ preservative, a 3 mL input volume was used, while for the neat samples, 2.1 mL of urine supplemented with 0.9 mL of PBS was used as an input volume (to ensure equal urine volumes in extraction). The cfTNA extractions were aliquoted in 25 µL aliquots for cell-free DNA analysis.

- Cellular pellet extraction

Samples were extracted in batches such that all corresponding pellets (baseline, time points, or post-freeze-thaw cycling) from a given pooled sample were extracted at the same time. Samples were removed from the −80 °C freezer and thawed at RT. The cellular pellets were extracted using the PowerFecal Pro DNA Kit (Qiagen, Hilden, Germany). 

#### 2.2.4. PCR Analysis

The extracted analytes were analyzed using the qPCR assays outlined in Table 1. All samples (baseline, time points, or post-freeze-thaw cycling) from a given target analyte and pooled urine samples were quantified on the same qPCR run. All samples, standards, and no template controls (NTC) were run in duplicate. For the 16S bacterial rDNA qPCR and TS143 qPCR assays, all FP samples were diluted 1:10 in nuclease-free water prior to the assay.

The human β-globin qPCR (cfDNA) reactions were performed using a C1000 Touch Thermal Cycler (Bio-Rad, Hercules, California, USA), while the 16S bacterial rDNA qPCR and TS143 qPCR reactions were performed on a Rotorgene RG-3000A/RG-6000 (Corbett), with the conditions described in Table 2. The average CT value for each sample was determined by taking the average of the qPCR replicates.

### 2.3. UAS™ Preservative Performance on Clinical Samples during Collection and Storage

#### 2.3.1. Sample Collection

This study is part of URODETECT (ClinicalTrials.gov identifier: NCT05453604) and was approved by the Ethical Committee of the Antwerp University Hospital (UZA) (EC filing: 20/10/115). Random midday, first-void urine samples were obtained at the Antwerp University Hospital and the University of Antwerp from four healthy female volunteers (HVFs), six healthy male volunteers (HVMs), seventeen pregnant women (PRW), five breast cancer patients (BCP), and five prostate cancer patients (PCP) after signing informed consents. All participants collected a first-void urine sample using Colli-Pee^®^ UAS™ FV-5040 (Novosanis, Wijnegem, Belgium).

#### 2.3.2. Experimental Set-Up

**Figure 2:**
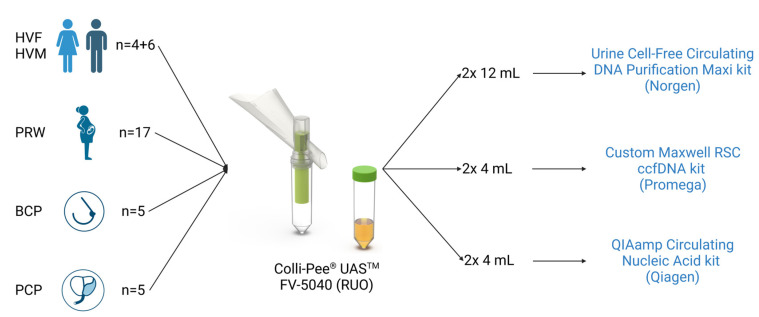
Experimental set-up of clinical study comparing different isolation methods—Abbreviations: BCP, breast cancer patients; HVF, healthy volunteer female; HVM, healthy volunteer male; PCP, prostate cancer patients; PRW, pregnant women. Created with BioRender.com.

Each collected sample was immediately centrifuged at 3000× *g* for 10 min, and the supernatants were divided into six aliquots (2 × 12 mL, 4 × 4 mL; Figure 2). From each sample, two aliquots were taken as technical replicates to account for method variance.

#### 2.3.3. Cell-Free DNA Extractions

Three commercially available cfDNA isolation kits were investigated: (i) the Urine Cell-Free Circulating DNA Purification Kit (Norgen Biotek Corp., Thorold, ON, Canada), which is column-based and developed to purify and concentrate high-quality, high-purity, and inhibitor-free cell-free circulating DNA. (ii) the Maxwell^®^ RSC Circulating DNA Purification Kit (Promega, Madison, WI, USA), which is magnetic beads-based and developed to purify fragmented DNA from human plasma with a protocol adapted for urine. (iii) the QIAamp^®^ Circulating Nucleic Acid Kit (Qiagen, Hilden, Germany), which is column- and vacuum-based and developed to purify and concentrate free-circulating DNA, RNA, miRNA, and viral nucleic acids from human plasma, serum, urine, or other cell-free body fluids.

#### 2.3.4. Analysis

The DNA concentration of each sample was determined using the Qubit^®^ dsDNA High Sensitivity Assay Kit (Thermo-Fisher Scientific, Waltham, MA, USA). Additionally, the sample DNA profile and cfDNA percentage were investigated using the Cell-Free DNA ScreenTape (Agilent, Santa Clara, CA, USA) for the Agilent 4150 TapeStation system. The DNA profile and cfDNA percentage were analyzed using Agilent TapeStation analysis software 4.1.1. cfDNA fragments are represented by a region ranging from 40–450 bp.

### 2.4. Statistical Analysis

Data is expressed as mean ± SEM unless otherwise indicated. All analyses were performed using GraphPad Prism (version 9.4.1, GraphPad Software Inc., La Jolla, CA, USA). All datasets were first explored for normality and outliers before the appropriate statistical test was applied. Statistical testing was performed using Welch’s T-test, Kolmogorov-Smirnov test, Kruskal-Wallis, Welch’s ANOVA test with a Dunnett’s T3 multiple testing correction, and a Factorial ANOVA for the factors “preservative” and “time” with a Tukey multiple testing correction, respectively. Data were considered significant at *p* < 0.05. Applied statistical analyses are indicated in the figure legend.

## 3. Results

### 3.1. Pre-Evaluation of the Need for Urine Preservation

**Figure 3:**
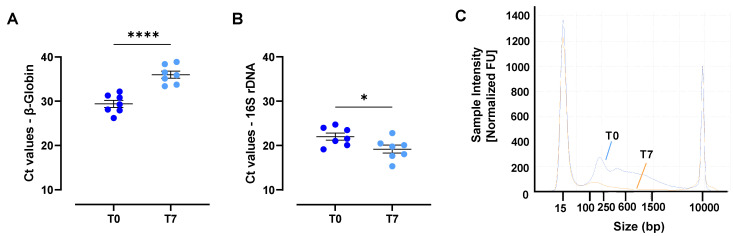
Pre-evaluation of the need for urine preservation—(**A**) human cell-free DNA presence; (**B**) bacterial growth; and (**C**) representative cell-free DNA profile of a female donor at baseline or after 7 days of storage at room temperature (20–26 °C). All experiments were performed with n = 7. For statistical analysis, Welch’s T-test was used (**A**,**B**). Statistical significance levels for comparison: * *p* ≤ 0.03; **** *p* ≤ 0.0001. Data are presented as mean ± SEM. Abbreviations: 16S rDNA, 16S bacterial DNA; T0, baseline; T7, 7 days.

Unpreserved urine samples stored at RT for 7 days showed a prominent decrease in human cfDNA (mean ∆Ct = 6.58) and a clear increase in bacterial cfDNA (mean ∆Ct = −2.83). Statistically significant differences were noticed for both human cfDNA (*p* < 0.0001; Figure 3A) and bacterial cfDNA (*p* = 0.0367; Figure 3B) after 7 days of storage at RT compared to baseline (Figure 3C). Overall, these results demonstrate bacterial growth in unpreserved urine as well as the loss of human cfDNA over time.

### 3.2. Evaluation of the UAS™ Preservative for Urine Storage

#### 3.2.1. Storage at Room Temperature for up to 14 Days

**Figure 4:**
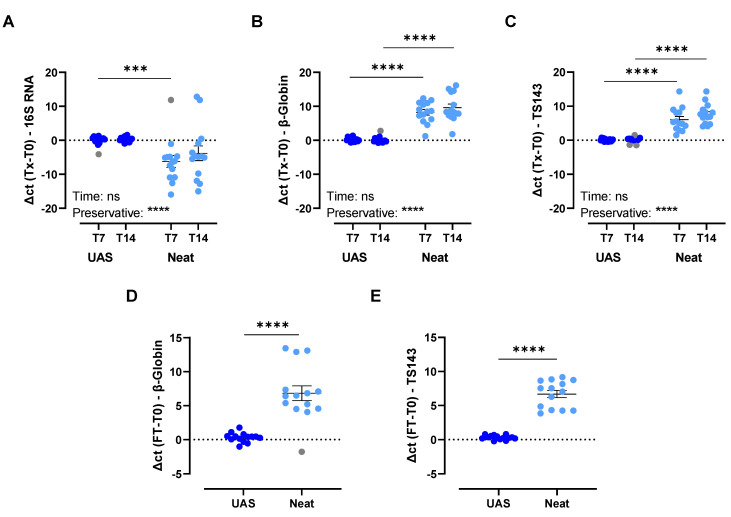
Performance of UAS™ preservative under different storage conditions—(**A**) microbial growth prevention; (**B**) cell-free DNA stability; and (**C**) host cellular integrity after 7 and 14 days of storage at room temperature (RT, 20–26 °C). All RT experiments were performed with n = 14 urine pools. Outliers were examined, and if identified, they were shaded in gray in the relevant graphs and removed from the statistical analysis: (**A**) two, (**B**) one, and (**C**) three outliers. Factorial ANOVA with a Tukey post-hoc test was performed for the factors “preservative” and “time” (**A**–**C**). The factor “preservative” was statistically significant in (**A**) *p* < 0.0001, (**B**) *p* < 0.0001, and (**C**) *p* < 0.0001. (**D**) cell-free DNA stability and (**E**) host cell integrity after freeze-thaw (FT) cycling (3×). All FT cycling experiments were performed with n = 14 urine pools. Outliers were investigated and, if identified, removed: (**D**) one outlier was removed from the statistical analysis and graphical representation. The Kolmogorov-Smirnov test (**D**) and Welch’s T-test (**E**) were performed. For all experiments, statistical significance levels for comparison are: *** *p* < 0.0002; **** *p* < 0.0001. Data are presented as mean ± SEM. Abbreviations: FT, freeze/thaw cycling; RT, room temperature; T0, baseline; T7, 7 days; T14, 14 days; TX, T7 or T14.

Different analytes were investigated to assess the overall performance of the UAS™ preservative for the storage of urine at RT for up to 7 and 14 days. Investigation into microbial growth prevention showed a statistically significant difference between samples preserved with UAS™ (∆Ct = 0.30) and neat samples (∆Ct = −7.64) after 7 days (*p* < 0.0002; Figure 4A). In addition, a comparison of cfDNA and host cell integrity indicated statistically significant differences between UAS™ preserved urine samples and neat samples after 7 days (cfDNA UAS™, ∆Ct = 0.18; cfDNA neat, ∆Ct = 8.25; host cell UAS™, ∆Ct = 0.07; host cell neat, ∆Ct = 6.05; *p* < 0.0001; Figure 4B) and 14 days storage at RT (cfDNA UAS™, ∆Ct = −0.01; cfDNA neat, ∆Ct = 9.61; host cell UAS™, ∆Ct = 0.33; host cell neat, ∆Ct = 7.68; *p* < 0.0001; Figure 4C). Moreover, the quality of cfDNA was checked by TapeStation analysis of cell-free nucleic acids extracted from a set of representative urine samples from Figure 4 stored for 7 and 14 days post-collection. No differences in fragmentation profiles were found between timepoints. Taken together, these results show an overall statistically significant inhibition of microbial growth and the preservation of cfDNA and host cell integrity when UAS™ is used as a preservative compared to neat urine samples stored at RT for up to 14 days.

#### 3.2.2. Impact of Simulated Shipping Conditions

Different analytes were investigated to assess the performance of UAS™ preservative as a urine preservative during simulated shipping conditions by freeze-thaw cycling. Three freeze-thaw cycles (−20 °C to +40 °C) were performed on each sample. A statistically significant difference (*p* < 0.0001) was observed between UAS™ preserved (∆Ct = 0.32) and neat (∆Ct = 7.49) urine samples related to cfDNA preservation (Figure 4D). Additionally, statistically significant differences between UAS™ preserved and neat urine samples for host cell integrity (UAS™, ∆Ct = 0.31; neat, ∆Ct = 6.68; *p* < 0.0001; Figure 4E) were observed. Taken together, these results show an overall statistically significant improvement in the preservation of cfDNA and host cell integrity when UAS™ is used as a preservative compared to neat urine samples after freeze-thaw cycling.

### 3.3. Evaluation of First-Void Urine Preserved with UAS™ and Compatibility with Three Commercially Available Isolation Methods for Urinary cfDNA

**Figure 5:**
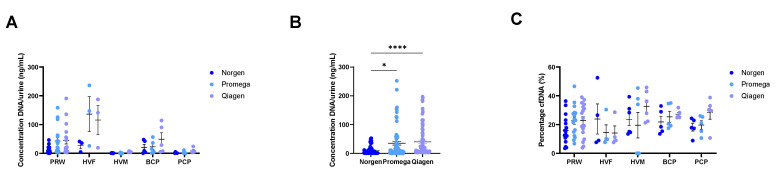
The UAS™ preservative is compatible with extraction methods based on different mechanisms as assed using clinical samples—(**A**) DNA concentration of each participant group and colored per isolation method; (**B**) DNA concentrations combined per isolation method; and (**C**) percentage cell-free DNA of each participant group and colored per isolation method. The isolations were performed with three different isolation methods (Norgen, Promega, and Qiagen) from healthy (HVF n = 4; HVM n = 6; PRW n = 17) and clinical (BCP n = 5; PCP n = 5) urine samples. Welch’s ANOVA test with Dunnett’s T3 correction (**A**) and Kruskal-Wallis tests (**B**,**C**) were performed. For all experiments, statistical significance levels for comparisons are: *, *p* < 0.03; and ****, *p* < 0.0001. Data are presented as mean ± SEM. Abbreviations: BCP, breast cancer patients; HVF, healthy female volunteers; HVM, healthy male volunteers; Norgen, Urine Cell-Free Circulating DNA Purification Kit; PCP, prostate cancer patients; Promega, Maxwell^®^ RSC Circulating DNA Purification Kit; PRW, pregnant women; Qiagen, QIAamp^®^ Circulating Nucleic Acid Kit.

The DNA concentration of the different samples ranged from 0.00 ng DNA/mL urine to 236.53 ng DNA/mL urine without statistically significant differences (Figure 5A); however, lower averages were found for the male samples (2.30 ± 2.33 ng DNA/mL urine for healthy male volunteers and 4.63 ± 3.66 ng DNA/mL urine for prostate cancer patients) compared to the female samples (98.43 ± 58.05 ng DNA/mL urine for healthy female volunteers, 33.28 ± 19.02 ng DNA/mL urine for pregnant women, and 31.04 ± 15.87 ng DNA/mL urine for breast cancer patients). Statistically significant differences were demonstrated between the Norgen isolation kit and the Promega (*p* < 0.03) and Qiagen (*p* < 0.0001) isolation kits, indicating that a higher amount of DNA per start volume of urine was obtained using the Promega and Qiagen isolation kits (Figure 5B). It is important to note that for Norgen, the starting volume of urine was higher, allowing fewer isolations to obtain more DNA. Furthermore, the cfDNA percentage of all participant types averaged around 20%, with no statistically significant differences between the participant types (Figure 5C). Additionally, no profound differences in sample DNA profiles were found between the urine samples of all different participant types or the isolation methods (Figure S1). Overall, these results suggest that all three commercially available isolation methods performed comparably based on DNA profiles and cfDNA percentages for the isolation of urinary cfDNA. Additionally, the UAS™ preservative is extraction-agnostic given its compatibility with three isolation methods based on different mechanisms.

## 4. Discussion

Urine is a promising emerging liquid biopsy; however, guidelines around pre-analytical parameters, including collection, preservation, and storage, are not yet well defined or standardized. Therefore, a novel preservative, UAS™, has been designed to prevent chemical and enzymatic degradation of nucleic acid and cellular lysis, as well as microbial growth. The performance of this recently developed and patented preservative [15], UAS™, was assessed in the current study for the preservation of urinary cfDNA and host cell integrity. First, a small pilot study was designed using urine from healthy volunteers to simulate a baseline experiment and demonstrate the importance of urine preservation. Thereafter, two studies were performed to demonstrate the preservation of urinary cfDNA and host cell integrity at RT for up to 14 days and after simulated transport conditions. Finally, the UAS™ preservative was used to collect samples from a patient cohort to show the applicability of UAS™ preservative in a clinical setting with individual samples. Together, these three different studies highlight the preservative value of UAS™, and its performance under various expected sample handling conditions, and its potential in a clinical setting. This work demonstrates the potential of Colli-Pee^®^ UAS™ in addressing the important issue of standardization of preanalytical workflows by allowing volumetric urine collection combined with optimized preservation, storage, and transportation conditions, thereby improving downstream processes. Given the expected heterogeneity in analyte concentrations between healthy and clinical samples, it would be valuable for future studies to further evaluate UAS™ preservative performance with large clinical cohorts.

### 4.1. Importance of Urinary Analyte Preservation

Standardization of the pre-analytical parameters for urine processing, such as urine collection, storage, and handling, is critical for the clinical usefulness of urinary biomarkers [17,18,19]. All these parameters may lead to water evaporation, host cellular lysis, nuclease activity, bacterial growth and/or contamination, and potentially changes in urine composition [20,21]. Preventing bacterial growth is important as it can cause the urine sample to reach higher turbidity, making the extraction and detection of specific analytes more difficult [22]. Preservation of host cell integrity is important to avoid host cell lysis and release of their cellular contents, including DNA, thereby preventing an accurate assessment of cfDNA. Urine contains potassium, calcium, magnesium, sodium, and zinc and has a pH between 5.0 and 7.0, providing a suitable environment for nuclease activity. DNase I, one of these DNA-hydrolyzing enzymes, has a more than 100-fold higher activity in urine compared to its activity in blood. DNase II is also present in urine, although its activity is 30 times lower than that of DNase I. On the other hand, RNA-hydrolyzing enzymes, including RNase I, RNase II, and phosphodiesterase I, are also present in urine. All these nucleic acid-hydrolyzing enzymes jeopardize the preservation of DNA and RNA fragments [9]. Our experiments confirmed prior findings that accurate analysis of urinary analytes requires prevention of microbial growth and that urinary analytes should be protected against nucleases and chemical degradation [4,5,9,12,13,14,23,24]. The addition of preserving agents, like ethylenediaminetetraacetic acid (EDTA), has been demonstrated to improve the stability of urinary analytes upon storage [4,13,17,18,19,20,24,25,26]. EDTA is a well-established chelating agent that binds the ions required for DNase activity. Consequently, adding EDTA inhibits nucleic acid hydrolyzing enzymatic activity and reduces DNA degradation [23]. EDTA is a frequently used preservative; however, it can only protect DNA [17,18,19,26]. Other preservatives have been frequently used for other analytes, e.g., guanidine thiocyanate preservatives for RNA [17] and boric acid or thymol preservatives for metabolites [20]. Currently, it appears that each analyte requires a different preservative. However, from a collection and processing point of view, the need for multiple reagents is not convenient for the continuously evolving multi-omics scene, emphasizing the need to validate an efficient preservative in urine that can preserve multiple analytes. Additionally, preservatives allow the storage of urine prior to analysis, whether due to home collection, sample shipment, or biobanking. The benefit of home collection for participants or patients is the comfort and privacy of a home environment to provide a sample without having to travel or visit a clinic; this in turn increases donor compliance and allows for easier recruitment of larger cohorts. The UAS™ preservative has been developed for preservation at room temperature, and the Colli-Pee^®^ device has been developed for the standardized volumetric collection and immediate preservation of urine while being user-friendly and suited for home collection.

### 4.2. Performance of UAS™ Preservative

The performance of UAS™ preservative as a urinary analyte preservative was examined in different experiments on the preservation of cfDNA and host cell integrity after (i) storage at room temperature for up to 14 days and (ii) freeze-thaw cycling. The room temperature experiment was designed to investigate the performance of UAS™ preservative under standard sample collection, storage, and handling conditions, while the freeze-thaw experiment was designed to be representative of transport conditions that will occur when samples are sent to the laboratory. First, UAS™ preservative was shown to prevent microbial growth and preserve cfDNA and host cell integrity, as urine samples preserved with UAS™ preservative showed statistically significantly improved results than neat urine samples after up to 14 days at room temperature and after freeze-thaw cycling (Figure 4). In this study, human cellular integrity was determined indirectly by the concentration of human DNA extracted from urine cellular pellets, and no direct measurements such as microscopy, flow cytometry, or fluorescent dyes were used. During sample storage, human cell lysis would compromise the availability of the cellular pellet after the centrifugation step, which would ultimately result in the loss of cellular DNA (leading to a change in the TS143 qPCR ∆Ct). These results further add to the applicability of the UAS^TM^ preservative in biobanking and batch extraction of urinary analytes from patients’ samples received in clinics at varying times. Another note about these experiments and results is the pooling of urine samples from different gender-equal individuals, as done in previous studies as well [20,27]. The experimental design combines urine samples from three female or male individuals to form one female or male, respectively, pooled urine sample, which removes the individualistic character of the samples. For these experiments, however, the benefits of pooling outweighed the need for the individualistic character of a particular sample. Since the experiments were performed using healthy volunteers, the individualistic nature of the urine samples was secondary to the pooling, which allowed for (i) an experimental design with higher volumes so that each condition was measured with the same pooled sample, (ii) a broad representation of the performance of UAS™ preservative on urine, and (iii) an evaluation independent of the parameters influencing a subject’s urine sample. For the clinical trial experiment, the samples were not pooled and kept as individual samples to maintain the uniqueness of each sample type since there were different cancers examined. 

### 4.3. Clinical Applicability of First-Void Urine Preserved with UAS™

In the URODETECT study, three commercially available isolation methods were tested and compared for their performance in isolating cfDNA from urine collected from different participant groups with Colli-Pee^®^ UAS™. This experiment led to two interesting conclusions: (i) cfDNA targets as urinary biomarkers and (ii) the compatibility of UAS™ preservative with different isolation methods. Firstly, cfDNA was investigated in different study populations: healthy female and male volunteers, pregnant women, and breast and prostate cancer patients. There were some differences in the DNA concentration between these groups; however, the percentage of cfDNA was relatively similar. This result emphasizes the ability to compare a healthy population to a diseased population and thereby the opportunity to explore cfDNA targets as biomarkers for prenatal and cancer diagnosis, screening, and monitoring of disease progression and recurrence [28]. Secondly, three isolation methods were examined: the Urine Cell-Free Circulating DNA Purification Maxi Kit [29], the QIAamp^®^ Circulating Nucleic Acid Kit [30], and the Maxwell^®^ RSC Circulating DNA Purification Kit [31], which are all based on different isolation mechanisms. Since our results show comparable performance for all three different isolation methods, it was demonstrated that UAS™, as a preservative, is compatible with and does not interfere with silicon-carbide technology, silica-membrane vacuum technology, or paramagnetic particle technology.

### 4.4. Strengths, Limitations, and Future Research

The strengths of our study are the test set-ups for investigating both the storage of urine at room temperature and under simulated transport conditions. This allowed assessing the performance of UAS™ for its preservation capabilities of urinary cfDNA and host cell integrity under all these conditions and preventing bacterial overgrowth. This is important for its applicability for at-home collection and shipment of samples to the laboratory. To enhance the clinical utility of urine for cancer research, it is considered a strength to have a volumetric urine collection device, Colli-Pee^®^, allowing for standardization and immediate mixing of urine with the UAS™ preservative to improve pre-analytical conditions and downstream processing.

Our study also has some limitations. First, the UAS™ performance studies were conducted on healthy volunteers only. Secondly, the study conducted in a clinical setting was on a small cohort of patient samples, and no targeted downstream analysis, such as PCR or NGS, was performed. The latter has important implications since Qubit analysis may overestimate the DNA concentration after isolation. Especially for the Qiagen isolation kit, because in this protocol carrier RNA is added, which can influence the Qubit DNA measurements.

Due to these strengths and limitations, future research is necessary to further elaborate on the potential of Colli-Pee^®^ UAS™ for the standardized and volumetric collection and preservation of first-void urine. In future research, it is warranted to (i) use patient samples to investigate the overall performance of UAS™; (ii) include larger patient cohorts to properly investigate the differences between groups; (iii) perform targeted downstream analysis; and (iv) investigate the potential of UAS™ for other urinary analytes such as extracellular vesicles, RNA, and proteins. Additionally, research is ongoing to investigate further improvements to the preservative: urine ratio, including (i) reducing the amount of liquid preservative in the collector tube and (ii) exploring possibilities for the creation of a powder/solid version of the preservative using lyophilization, spray drying, and other technologies. If further elucidated, the potential of Colli-Pee^®^ UAS™ could enable individuals to collect a urine sample in the comfort of their own home and send the sample to the laboratory, all in a more standardized manner. That would all help to increase cancer screening coverage and provide more comfort to cancer patients during their treatment and disease follow-up.

## 5. Conclusions

Urine as a liquid biopsy has the potential to become a game changer in personalized cancer care, allowing for its use for primary and secondary prevention, diagnosis, treatment response measurement, and detection of recurrence. Besides the need for clinical validity and utility, there is also an urgent need for analytical validity. The current study clearly tackled the important issue of lack of standardization of preanalytical workflows and demonstrated the potential of Colli-Pee^®^ UAS™ as a suitable urine collection and preservation method allowing for standardization in collecting, preserving, and analyzing. Specifically, the UAS™ preservative allows for accurate and clinically translatable analysis of urinary analytes, prevention of bacterial growth and analyte degradation, as well as preservation of host cell integrity and cfDNA during pre-analytical sample storage and handling, which is deemed critical. The UAS™ preservative also showed statistically significant improvement in the preservation of host cell integrity and cfDNA during freeze-thaw cycling. The agnostic nature of the UAS™ preservative was demonstrated by its compatibility with commercially available isolation methods based on different mechanisms using clinical samples. Additionally, the study demonstrates the applicability of the first-void at-home urine collection device Colli-Pee^®^ UAS™ by Novosanis as a standardized, volumetric tool to collect urine and immediately preserve urinary analytes for oncology applications.

## Figures and Tables

**Table 1:** Overview of qPCR assays for the different targets—Summary of the different qPCR and RT-qPCR assays that were used for the analysis of the different analytes. The human β-globin qPCR assay was used to determine cfDNA stability, the 16S bacterial rDNA qPCR for microbial growth prevention, and the TS143 qPCR for host cell integrity. The commercially available products used are from Bio-Rad (Hercules, CA, USA) or Thermo-Fisher Scientific (Waltham, MA, USA). Abbreviations: ACTB, beta-actin; cfDNA, cell-free DNA; GAPDH, glyceraldehyde 3-phosphate dehydrogenase; TS143, thymidylate synthase gene with an expected qPCR product size of 143 bp.

PCR Assay	Master Mix	Forward Primer	Reverse Primer
Human β-globin qPCR [16]	2X iTaq Universal SYBR Master mix (Bio-Rad)	5′-ACACAACTGTGTTCACTAGC-3′	5′-CAACTTCATCCACGTTCACC-3′
16S qPCR	Taq DNA Polymerase + Syto 9-based in-house master mix	5′-ATTACCGCGGCTGCTGG-3′	5′-CCTACGGGAGGCAGCAG-3′
TS143 qPCR		5′-GCCCTCTGCCAGTTCTA-3′	5′-GCCCTCTGCCAGTTCTA-3′

**Table 2:** Overview of the cycling conditions of each PCR assay—Overview of the amplification conditions used for each qPCR and RT-qPCR assay (see Table 1). Abbreviations: 16S, 16S bacterial rDNA; TS143, thymidylate synthase gene with an expected qPCR product of 143 bp.

Steps	Human β-Globin qPCR			16S qPCR			TS143 qPCR		
Initial heat activation	5 min	95 °C	1×	2 min	95 °C	1×	5 min	95 °C	1×
Denaturation	20 s	95 °C	45×	30 s	95 °C	35×	20 s	95 °C	35×
Annealing/Extension	30 s	56 °C		20 s	55 °C		20 s	55 °C	
				20 s	72 °C		30 s	72 °C	

## Data Availability

The data presented in this study are available on request from the corresponding author. The data are not publicly available since most experiments are performed in a corporate context, and these databases are not publicly available. However, when requested, data can be shared.

## Supplementary Materials

The following supporting information can be downloaded at: https://www.mdpi.com/article/10.3390/cancers15123119/s1. Figure S1: Cell-free DNA profiles using Colli-Pee^®^ UAS™ on clinical samples.

## Abbreviations

BCP	Breast cancer patient
cDNA	Copy DNA
cfDNA	Cell-free DNA
cfTNA	Cell-free total nucleic acids
EDTA	Ethylenediaminetetraacetic acid
FP	Female pooled urine samples
HVF	Healthy volunteer female
HVM	Healthy volunteer male
ISEV	International Society for Extracellular Vesicles
lncRNA	Long non-coding RNA
miRNA	microRNA
MP	Male pooled urine samples
NTC	Non-template control
PCP	Prostate cancer patient
PRW	Pregnant women
RT	Room temperature
UZA	Antwerp University Hospital

## References

[1] Sung H., Ferlay J., Siegel R.L., Laversanne M., Soerjomataram I., Jemal A., Bray F. (2021). Global Cancer Statistics 2020: GLOBOCAN Estimates of Incidence and Mortality Worldwide for 36 Cancers in 185 Countries. CA Cancer J. Clin..

[2] Tsui N.B., Jiang P., Chow K.C., Su X., Leung T.Y., Sun H., Chan K.C., Chiu R.W., Lo Y.M. (2012). High resolution size analysis of fetal DNA in the urine of pregnant women by paired-end massively parallel sequencing. PLoS ONE.

[3] Di Meo A., Bartlett J., Cheng Y., Pasic M.D., Yousef G.M. (2017). Liquid biopsy: A step forward towards precision medicine in urologic malignancies. Mol. Cancer.

[4] Larsen L.K., Lind G.E., Guldberg P., Dahl C. (2019). DNA-Methylation-Based Detection of Urological Cancer in Urine: Overview of Biomarkers and Considerations on Biomarker Design, Source of DNA, and Detection Technologies. Int. J. Mol. Sci..

[5] Kretschmer-Kazemi Far R., Frank K., Sczakiel G. (2021). Sampling, Logistics, and Analytics of Urine for RT-qPCR-based Diagnostics. Cancers.

[6] Jordaens S., Zwaenepoel K., Tjalma W., Deben C., Beyers K., Vankerckhoven V., Pauwels P., Vorsters A. (2023). Urine biomarkers in cancer detection: A systematic review of preanalytical parameters and applied methods. Int. J. Cancer.

[7] Dermody S.M., Bhambhani C., Swiecicki P.L., Brenner J.C., Tewari M. (2022). Trans-Renal Cell-Free Tumor DNA for Urine-Based Liquid Biopsy of Cancer. Front. Genet..

[8] Su Y.H., Wang M., Brenner D.E., Ng A., Melkonyan H., Umansky S., Syngal S., Block T.M. (2004). Human urine contains small, 150 to 250 nucleotide-sized, soluble DNA derived from the circulation and may be useful in the detection of colorectal cancer. J. Mol. Diagn..

[9] Bryzgunova O.E., Laktionov P.P. (2015). Extracellular Nucleic Acids in Urine: Sources, Structure, Diagnostic Potential. Acta Nat..

[10] Peng M., Chen C., Hulbert A., Brock M.V., Yu F. (2017). Non-blood circulating tumor DNA detection in cancer. Oncotarget.

[11] Lu T., Li J. (2017). Clinical applications of urinary cell-free DNA in cancer: Current insights and promising future. Am. J. Cancer Res..

[12] Vorsters A., Micalessi I., Bilcke J., Ieven M., Bogers J., Van Damme P. (2012). Detection of human papillomavirus DNA in urine. A review of the literature. Eur. J. Clin. Microbiol. Infect Dis..

[13] Bosschieter J., Bach S., Bijnsdorp I.V., Segerink L.I., Rurup W.F., van Splunter A.P., Bahce I., Novianti P.W., Kazemier G., van Moorselaar R.J.A. (2018). A protocol for urine collection and storage prior to DNA methylation analysis. PLoS ONE.

[14] Locke W.J., Guanzon D., Ma C., Liew Y.J., Duesing K.R., Fung K.Y.C., Ross J.P. (2019). DNA Methylation Cancer Biomarkers: Translation to the Clinic. Front. Genet..

[15] Arora A. (2021). Stabilizing Composition and Method for Preserving a Bodily Fluid. Patent.

[16] Jung M., Klotzek S., Lewandowski M., Fleischhacker M., Jung K. (2003). Changes in concentration of DNA in serum and plasma during storage of blood samples. Clin. Chem..

[17] Lin S.Y., Linehan J.A., Wilson T.G., Hoon D.S.B. (2017). Emerging Utility of Urinary Cell-free Nucleic Acid Biomarkers for Prostate, Bladder, and Renal Cancers. Eur. Urol. Focus.

[18] Cannas A., Kalunga G., Green C., Calvo L., Katemangwe P., Reither K., Perkins M.D., Maboko L., Hoelscher M., Talbot E.A. (2009). Implications of storing urinary DNA from different populations for molecular analyses. PLoS ONE.

[19] Zhou Q., Liu F., Guo L., Chen R., Yuan X., Li C., Shu L., Liu H., Zhou Y., Wu Y. (2021). A novel urine cell-free DNA preservation solution and its application in kidney transplantation. Nephrology.

[20] Wang X., Gu H., Palma-Duran S.A., Fierro A., Jasbi P., Shi X., Bresette W., Tasevska N. (2019). Influence of Storage Conditions and Preservatives on Metabolite Fingerprints in Urine. Metabolites.

[21] Kouri T., Vuotari L., Pohjavaara S., Laippala P. (2002). Preservation of urine for flow cytometric and visual microscopic testing. Clin Chem.

[22] Thongboonkerd V., Saetun P. (2007). Bacterial overgrowth affects urinary proteome analysis: Recommendation for centrifugation, temperature, duration, and the use of preservatives during sample collection. J. Proteome Res..

[23] Barra G.B., Santa Rita T.H., de Almeida Vasques J., Chianca C.F., Nery L.F., Santana Soares Costa S. (2015). EDTA-mediated inhibition of DNases protects circulating cell-free DNA from ex vivo degradation in blood samples. Clin. Biochem..

[24] Vorsters A., Van den Bergh J., Micalessi I., Biesmans S., Bogers J., Hens A., De Coster I., Ieven M., Van Damme P. (2014). Optimization of HPV DNA detection in urine by improving collection, storage, and extraction. Eur. J. Clin. Microbiol. Infect. Dis..

[25] Eisinger S.W., Schwartz M., Dam L., Riedel S. (2013). Evaluation of the BD Vacutainer Plus Urine C&S Preservative Tubes compared with nonpreservative urine samples stored at 4 degrees C and room temperature. Am. J. Clin. Pathol..

[26] Li P., Ning J., Luo X., Du H., Zhang Q., Zhou G., Du Q., Ou Z., Wang L., Wang Y. (2019). New method to preserve the original proportion and integrity of urinary cell-free DNA. J. Clin. Lab. Anal..

[27] Hoppin J.A., Ulmer R., Calafat A.M., Barr D.B., Baker S.V., Meltzer H.M., Ronningen K.S. (2006). Impact of urine preservation methods and duration of storage on measured levels of environmental contaminants. J. Expo. Sci. Environ. Epidemiol..

[28] Bax C., Lotesoriere B.J., Sironi S., Capelli L. (2019). Review and Comparison of Cancer Biomarker Trends in Urine as a Basis for New Diagnostic Pathways. Cancers.

[29] Norgen Biotek Corp Urine Cell-Free Circulating DNA Purification Kit: Protocol. https://norgenbiotek.com/product/urine-cell-free-circulating-dna-purification-maxi-kit.

[30] Qiagen QIAamp Circulating Nucleic Acid Kit: Handbook. https://www.qiagen.com/us/products/discovery-and-translational-research/dna-rna-purification/dna-purification/cell-free-dna/qiaamp-circulating-nucleic-acid-kit/?catno=55114.

[31] Promega. Maxwell^®^ RSC ccfDNA Plasma Kit. https://be.promega.com/products/nucleic-acid-extraction/genomic-dna/maxwell-rsc-ccfdna-plasma-kit/?catNum=AS1840#protocols.

