# Supplementary material for: UAS™—A Urine Preservative for Oncology Applications
Source: Cancers (Basel). 2023 Jun 8;15(12):3119. doi: 10.3390/cancers15123119 (PMC10296352; doi:10.3390/cancers15123119)
Supplement: Supplementary file 1 [file cancers-15-03119-s001.zip › cancers-2354093-supplementary.pdf]

Supplementary materials

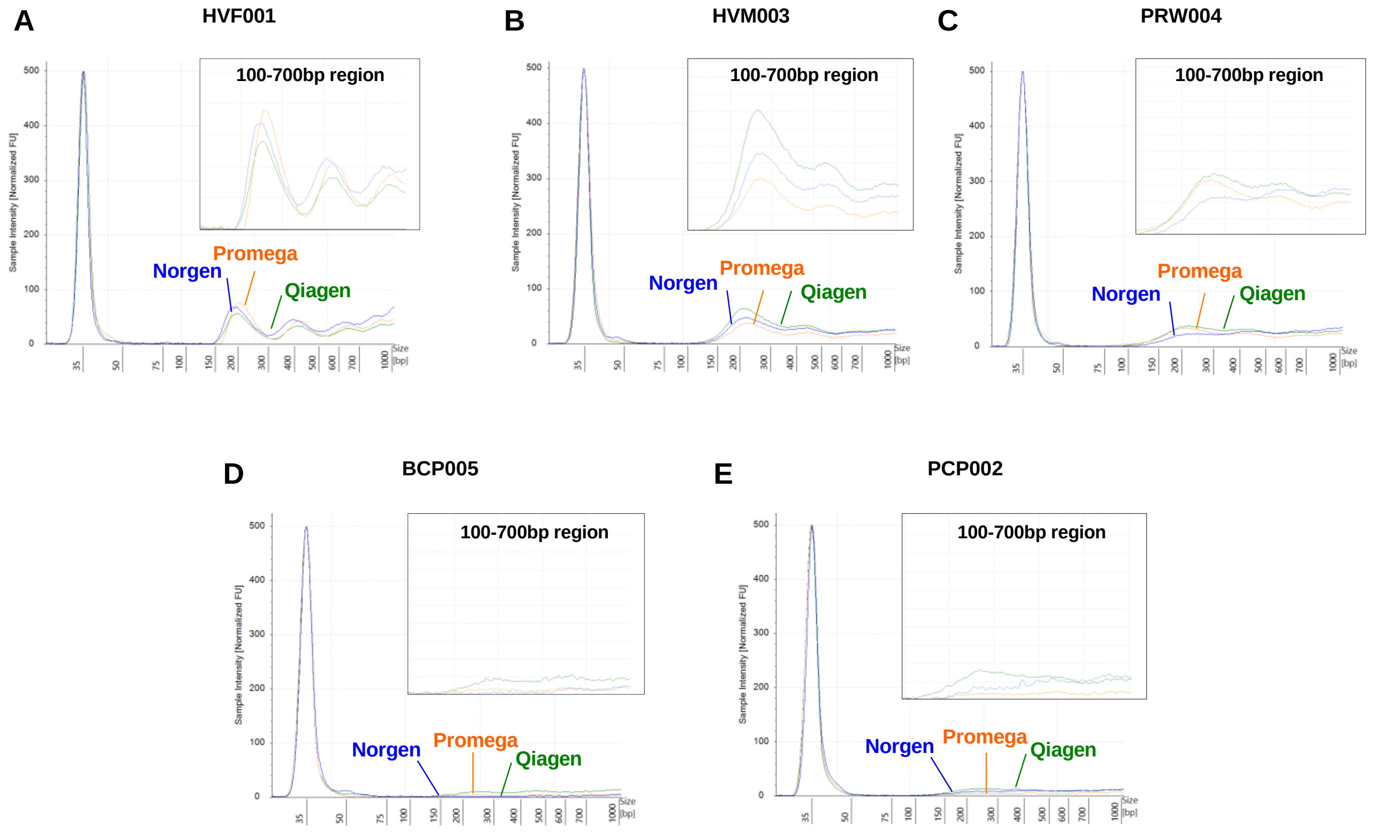

Figure S1: cell-free DNA profiles using Colli-Pee® UAS™ on clinical samples - Representative cfDNA profile of a urine sample from a healthy female volunteer (A), a healthy male volunteer (B), a pregnant woman (C), a breast cancer patient (D) and, a prostate cancer patient (E). Abbreviations: BCP, breast cancer patients; cfDNA, cell-free DNA; HVF, healthy female volunteers; HVM, healthy male volunteers; Norgen, Urine Cell-Free Circulating DNA Purification Kit; PCP, prostate cancer patients; Promega, Maxwell® RSC Circulating DNA Purification Kit; PRW, pregnant women; Qiagen, QIAamp® Circulating Nucleic Acid Kit.
